# Skeletal Anomalies in Senegalese Sole (*Solea senegalensis*, Kaup) Fed with Different Commercial Enriched *Artemia*: A Study in Postlarvae and Juveniles

**DOI:** 10.3390/ani11010022

**Published:** 2020-12-24

**Authors:** Ana Manuela de Azevedo, Ana Paula Losada, Isabel Ferreiro, Ana Riaza, Vanesa Losada, Tommaso Russo, Clara Boglione, Sonia Vázquez, María Isabel Quiroga

**Affiliations:** 1 Department of Anatomy, Animal Production and Clinical Veterinary Sciences, Faculty of Veterinary Science, Campus Universitario S/N, University of Santiago de Compostela, 27002 Lugo, Spain; anapaula.losada@usc.es (A.P.L.); sonia.vazquez@usc.es (S.V.); misabel.quiroga@usc.es (M.I.Q.); 2 Biology Department, Ghent University, Ledeganckstraat 35, 9000 Ghent, Belgium; 3 Stolt Sea Farm, Edificio Quercus, C/Letonia n.° 2, 15701 Santiago de Compostela, Spain; i.ferreiro@stolt.com (I.F.); a.riaza@stolt.com (A.R.); 4 ANFACO-CECOPESCA, Estrada Colexio Universitario, 16, 36310 Vigo, Spain; vlosada@anfaco.es; 5 Department of Biology, University of Rome Tor Vergata, Via della Ricerca Scientifica, 00133 Rome, Italy; Tommaso.Russo@Uniroma2.it (T.R.); boglione@uniroma2.it (C.B.); 6 Aquaculture Institute, Campus Vida, Rúa de Constantino Candeira, 15705 Santiago de Compostela, Spain

**Keywords:** skeletal anomalies, Senegalese sole (*Solea senegalensis*), enrichment products, rearing conditions

## Abstract

**Simple Summary:**

Enrichment products for *Artemia* spp. metanauplii are commonly used to enhance the nutritional quality of this live prey offered to fish during conventional larval feeding. However, there are few reports on the influence of such enrichments on the development of skeletal anomalies in Senegalese sole, a major problem for this flatfish aquaculture. This study evaluated the frequency of vertebral anomalies in postlarvae and juvenile Senegalese sole fed with *Artemia* spp. metanauplii enriched with four commercial products (EA, EB, EC, and ED) in a fish farm. The results show a high percentage of individuals with skeletal anomalies in every dietary group. Some types of anomalies were very frequent in all diet-age groups, indicating the presence of a common trend or mainstay of vertebral deformities. Despite some variations in the frequency of anomalies among diets, it was not possible to establish a clear effect of the enrichment products on the development of vertebral deformities at both rearing stages, probably for the “masking effect” of other rearing conditions. The multivariate statistical technique, as the correspondence analysis, indicated a different anomaly pattern among ages, where bone adaptative responses may be implied.

**Abstract:**

The high incidence of skeletal anomalies in Senegalese sole (*Solea senegalensis*) still constitutes a bottleneck constraining its production. There are diverse commercially available products for the enrichment of live preys, but few reports of their influence on skeletogenesis in Senegalese sole. This study evaluated the presence of vertebral anomalies in postlarvae and juvenile Senegalese sole fed with *Artemia* spp. metanauplii enriched with four commercial products (EA, EB, EC, and ED) in a fish farm. The most frequent alterations consisted of deformations of the neural/haemal arches and spines and fusions and deformations of hypurals, epural, or parhypural. The correspondence analysis ordered fish from each age in separated semiaxis, indicating the presence of different anomaly patterns for the two sampled stages. The results showed only very light changes in the frequency of vertebral abnormalities among tested enrichment products, i.e., individuals from EC and EA lots displayed less vertebral body anomalies and/or vertebral column deviations at 31 and 105 days after hatching, respectively. The existence of a large shared malformation pattern in all the experimental groups leads to impute to the rearing conditions as the main driving factor of the onset of such group of anomalies, probably masking some dietary effect.

## 1. Introduction

Senegalese sole (*Solea senegalensis*, Kaup) is a marine flatfish species with an increasing production over the last few years in Europe [1]. However, skeletal anomalies still represent one of the major limitations to its exploitation in large-scale aquaculture [2,3,4]. Several reports, both in experimental and in industrial conditions, showed an incidence from 44% to 100% of deformed individuals [2,5,6,7]. Studies in diverse cultured fish species showed the influence of environmental, nutritional, genetic, and many other factors on the development of skeletal deformities [8,9]. Nutrition plays an important role during the early stages of development [10]. In particular, dietary imbalances can provoke impairments in skeletogenesis and bone metabolism, resulting in skeletal abnormalities [11,12]. Nutrients like vitamins A, C, D, and K, minerals as calcium and phosphorus, and others as some fatty acids are related to the development of bone disorders [3,12,13,14,15].

Metanauplii of *Artemia* spp. are ready-to-use live prey for larval feeding but “nutritional enrichment” is largely applied in order to enhance their fatty acid composition [16,17]. Nowadays, there are diverse commercial products for the enrichment of *Artemia* spp. [4]. Their differences consist in the physical form, nutritional content, or ingredients [4], and in the composition of live prey that can vary according to culture/enrichment conditions [18]. Enriched *Artemia* spp. metanauplii are commonly incorporated into the sole diet during larval rearing [4,11,19]. However, there are few reports of the influence of the enrichments on the development of bone deformities in reared Senegalese sole, and especially in juveniles. In this sense, the study of Boglino et al. [4] showed no significant effect of the analyzed commercial enriching products in the total incidence of skeletal anomalies in experimentally reared larvae. 

The aim of this study was to evaluate the effects of different commercial products used for enriching *Artemia* spp. metanauplii on the skeletal anomalies in postlarvae and juvenile Senegalese sole.

## 2. Materials and Methods 

### 2.1. Rearing Protocol

The protocol and procedures employed in this experiment followed the European Directive 2010/63/EU.

Around 25,000 Senegalese sole larvae from a single egg batch (broodstock from Stolt Sea Farm) were reared in the experimental larval rearing facility of Stolt Sea Farm in Merexo, A Coruña (Spain). Genetic tests demonstrated that the offspring came from a single female and male. At one day after hatching (dah), larvae were randomly stocked in eight cylindrical tanks of 155 L with an open flow system, at an initial density of 20 larvae L^−1^. The number of fish in each tank was estimated by volumetric counting. The rearing procedure was identical for every tank. Water was daily renewed, starting from 4% per hour to 50% at the end of larval rearing in each tank; pH was the same as the sea water pH in the Atlantic Ocean (8.0–8.2) and photoperiod was 12 h light: 12 h dark. On day 15, all the fish exhibited benthic life and were transferred to 25 L cylindrical tanks. A daily individual counting for dead fish was initiated at 15 days after hatching (dah). The number of individuals in each tank at this point was calculated considering the number of fish in each tank at the end of the experiment, the number of dead fish per day, and the number of sampled fish (Table 1). At 28 dah, specimens were relocated in eight squared tanks of 130 L. During the experiment, water temperature and dissolved oxygen were, respectively, 19.2 ± 0.3 °C and 6.9 ± 0.8 ppm: mean ± standard deviation (SD).

Larvae feeding regime is depicted in Table 2. After feeding the larvae with rotifers (*Brachionus plicatilis*) enriched with microalgae (*Isochrysis galbana*), newly hatched *Artemia* spp. nauplii were supplied to larvae from 5 to 8 dah, at increasing density. Four different commercial products (EA, EB, EC, ED) (manufacture’s labeled nutritional information for each enrichment is provided in Table 3) were used to the enrichment of *Artemia* spp. metanauplii, according to the fish farm protocol and following specific manufactures instructions. Each enriched live prey diet was used to feed fish from two separated tanks (EA-1, EA-2; EB-1, EB-2; EC-1, EC-2; ED-1, ED-2, respectively). Enriched *Artemia* metanauplii were supplied to larvae at gradually increasing density, adjusted according to the increase of weight of the larvae. Commercial inert weaning diet was offered at regular intervals 10 to 20 times a day with automatic feeders from day 31 to 38, until the total withdrawal of live feed. Fish were fed commercial inert feed until the end of the experiment at 105 dah. 

### 2.2. Biochemical Analysis

Basic biochemical analysis of elemental nutrient profiles of prey and larvae was performed. For each enrichment diet, 15 g of *Artemia* metanauplii were collected at day 13 of larval rearing. At 29 dah, 300 postlarvae per experimental tank were sampled. All samples were cryopreserved in liquid nitrogen at −80 °C until posterior analysis at ANFACO-CECOPESCA (Vigo, Spain). The wet weight (*ww*) content of vitamins A (retinol) and C (mg kg^−1^), calcium, and phosphorous (g kg^−1^) was determined in live prey and postlarvae. Vitamin D_3_ (cholecalciferol) was quantified in µg kg^−1^ *ww* in every sample. Ratio Ca/P was then calculated. The fatty acid determination was performed in terms of percentage of the total of fatty acids (% TFA), focusing on polyunsaturated fatty acids (PUFA), in particular, n-3 and n-6 PUFA, eicosapentaenoic acid (EPA, 20:5n-3), and docosahexaenoic acid (DHA, 22:6n-3). DHA/EPA and (n-3)/(n-6) ratios were computed. 

Fat-soluble vitamins (A and D) were analyzed by HPLC after prior alkaline hydrolysis. Saponification and vitamin extraction procedures were adapted from Salo-Väänänen et al. [20]. The residue was filtered in a 0.45 µm filter prior to HPLC analysis. A HPLC Waters^®^ 2695 system was used equipped with a Sunfire Pro Silica column (Waters^®^, Milford, CT, USA), a photodiode array (PDA) detector (Waters^®^, Milford, CT, USA), and a fluorescence detector (Waters^®^, Milford, CT, USA). The analysis ran at a flow rate of 1.5 mL min^−1^, showing a retention time of 37.77 min. For vitamin D_3_ (cholecalciferol) detection, the flow rate was 1 mL min^−1^, and the obtained retention time was 38.17 min. Regarding vitamin C analysis, each sample was prepared according to Oruña-Concha et al. [21]. The supernatant was filtered by a porous filter of 0.2 µm and then by a syringe filter of 0.22 µm previously to HPLC analysis. A HPLC Waters^®^ 2695 system was used equipped with a YMC-Pack Pro C18 column (YMC™, Kyoto, Japan) and a PDA detector (Waters^®^, Milford, CT, USA). The HPLC ran at a flow rate of 0.5 mL min^−1^ and the retention time was 30 min. The concentration of vitamins A, C, and D_3_ in each sample was then calculated taking into account the calibration curve with standard solutions of retinol, L-ascorbic acid, and cholecalciferol, respectively. The techniques were validated taking into account the accuracy, precision, quantification limit, selectivity, specificity, and linearity of the method.

The identification and quantification of Ca and P was conducted in an inductively coupled plasma optical emission spectrometer (ICP-OES) VISTA MPX (Agilent^®^, Santa Clara, CA, USA) using between 0.5 g and 1 g of sample.

For fatty acid determination, 1 g of each sample was used. Total lipids were extracted in chloroform:methanol (1:2, v:v) using the method of Bligh and Dyer [22]. Fatty acid separation and identification were performed using the method of Sato and Murata [23] by means of gas–liquid chromatography using a gas chromatograph equipped with a flame ionization detector (FID) 6890 N (Agilent^®^, Santa Clara, CA, USA). 

### 2.3. Skeletal Anomaly Evaluation

Senegalese sole from each tank were randomly sampled at 31 dah and 105 dah (Table S1) for meristic counts and skeletal anomaly detection. Fish groups were annotated according to the respective age, enrichment product, and tank replicate (Figure S1). All sampled fish were sacrificed using an overdose of Tricaine methanesulfonate (MS-222, Sigma-Aldrich, Madrid, Spain). Specimens were fixed in 10% buffered formalin and stained using a modified double staining technique for cartilage and bone [24,25]. Standard length (StL) and standard height (StH) were measured at 31 dah and 105 dah. StL is the distance between the rostral end of the skull and the end of hypurals. StH comprised the higher length between the ventral and dorsal aspect of the fish, without taking into account the fins rays. The ratio StL/StH was computed. At the first sampling point, measurements (mm) on fixed samples were performed using a binocular model Olympus^®^ (Barcelona, Spain) SZX16 coupled to a digital camera (Olympus^®^ DP72, Barcelona, Spain) and an image processing program, ImageJ 1.50i [26]. Stained individuals were observed by both sides using a binocular model Olympus^®^ SZX16 (Barcelona, Spain). In the vertebral column, three anatomical regions were considered: abdominal vertebrae, caudal vertebrae, and caudal complex, according to Gavaia et al. [2]. For every region, the number of vertebrae was counted. Each vertebra and caudal complex element were evaluated for anomaly detection of the parapophyses, haemal arches and spines, neural arches and spines, and vertebral bodies, as described in previous studies [6]. Completely fused vertebrae were considered as one single vertebra in meristic counts if the union presented the same appearance of one single vertebra, but were registered as fusions in anomaly assessment. The type and number of anomalies were registered in all individuals. The typology of anomalies recorded according to the anatomical region is shown in Table S2. In this paper, we grouped the different anomaly typologies under 22 categories, one for each of the considered skeletal elements and region, i.e., category C-H consists of 6 anomaly typologies affecting the caudal haemal arches and spines. 

### 2.4. Statistics

For each tank, the daily survival rate was calculated from 15 dah to both sampling stages. Similarities in data distribution of measurements (StL, StH, and StL/StH) among replicate diets at 31 and 105 dah were assayed with a Kolmogorov-Smirnov test. The Pearson’s chi-square test was used to check for differences among replicates in the survival at both stages. Main descriptive statistical evaluation was performed on survival rate and biometric data: mean, standard error of the mean (SEM), median, minimum, and maximum. Kruskal-Wallis tests were applied to detect significant differences in the distribution of the daily survival rate and biometric values among dietary groups. Pairwise Mann–Whitney comparison tests (with Bonferroni corrections) were performed in case of finding significant differences (*p* < 0.05). Meristic counts were also expressed by means of the percentage of fish displaying the median number of vertebrae. 

For the two sampling points and for each tank it was calculated: the incidence of individuals displaying at least one anomaly and the percentage of individuals with vertebral body anomalies (VBA) and/or vertebral column deviations (VCD); the frequency of skeletal abnormalities; the percentage of VBA and/or VCD in the total number of anomalies of each lot and the average number of anomalies per affected fish (median ± range). Differences in the frequencies of specimens showing VBA and/or VCD among diet groups were assessed by means of an chi-square test of independence.

Similar to the procedure described in Prestinicola et al. [27], two matrices were elaborated. First, a binary matrix (BM) showed the presence (1 = at least one anomaly) or absence (0) of each anomaly category for each individual. The binary variable “absence of anomalies” (ABS) (1 = absence of anomalies; 0 = presence of at least one anomaly) was added to include soles without anomalies in the analysis. The second matrix (frequency matrix, FM) displayed the relative frequency of individuals for each anomaly category (and ABS) and diet-tank. A Correspondence Analysis (CA) was performed on FM of both developmental stages to organize data seeking for any relationship between anomaly categories, diet-tank, and age. CA was performed using RStudio^®^ version 1.2.5019 software [28] (RStudio Team 2019) with R version 3.6.2 [29] (R Core Team 2019). Descriptive statistics were calculated with Microsoft Office 365 Excel^®^. All the other statistical analyses were performed with Past version 2.17 [30].

## 3. Results

The number of individuals in each tank at 15 dah is shown in Table 1. If the volume of water in the tanks is considered (25 L), some differences in densities emerged among some replicates and groups: the range was comprised of 69 (ED-1) and 108 (EA-1) postlarvae/L. At 28 dah, the densities diminished considerably in all tanks due to the transfer of fish to bigger tanks. Still, some differences were found in the tank density until the end of the experimental rearing (105 dah), ranging from 9 (ED-1) to 16 (EA-1) postlarvae/L (Table S3). At 31 dah, eye migration was complete in all the sampled specimens. 

### 3.1. Biochemical Analysis

Metanauplii nutritional composition is summarized in Table 4. Nutritional values were comparable among diets. However, EB metanauplii showed a remarkably higher amount of Vit. D_3_. In turn, EC diet presented increased levels of DHA and DHA/EPA. Biochemical data of Senegalese sole at 30 dah are shown in Table 5. Fish from diet EB showed high amounts of Vit. D_3_ and (n-6) PUFA and EC-2 group displayed the highest DHA content. ED-1 and ED-2 groups showed an increased level of (n-3)/(n-6) PUFA. 

### 3.2. Survival Rate and Measurements

Kolmogorov-Smirnov tests for both StL and StH revealed significant differences between the two replicates of diets EC and ED at 31 dah. Fish at 105 dah did not present different measurement distributions between replicates. Differences among replicates in the survival at 105 dah were detected using the Pearson’s chi-square test. Therefore, replicates were merged into the respective four dietary groups except for EC and ED at 31 dah and ED at 105 dah, which were treated separately (31 EC-1, 31 EC-2, 31 ED-1, 31 ED-2, 105 ED-1, 105 ED-2). Thus, the groups of fish were arranged as in Table 6.

**Figure 1:**
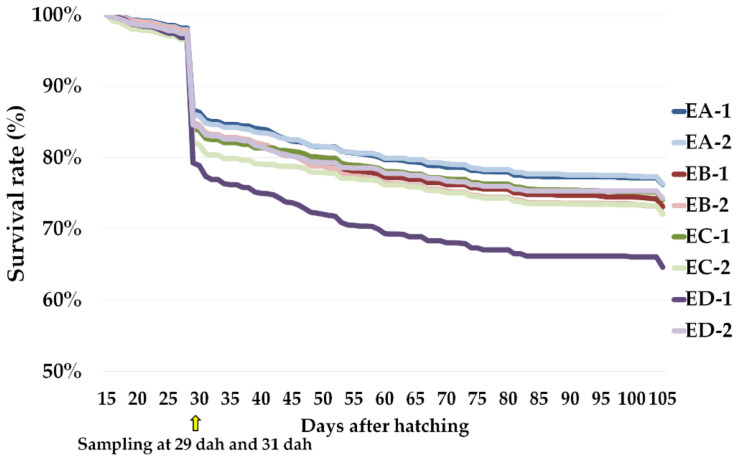
Survival rate (%) for each replicate (EA-1, EA-2, EB-1, EB-2, EC-1, EC-2, ED-1, ED-2) from 15 to 105 days after hatching.

Daily survival rate (%) for each tank from 15 dah to 105 dah is displayed in Figure 1 and Table S4 and it was comparable among tanks. Table 7 displays descriptive statistics for daily survival rate, StL, StH and StL/StH for each diet-tank group. Survival rate was comparable in 31 dah groups, although 105 EA and 105 ED-1 showed a higher and lower survival rate, respectively. In general, 31 EC-2 and 31 ED-1 were significantly bigger (both StL and StH) than 31 EB and 31 ED-2 (Table 7). These differences disappeared at 105 dah. 105 EA and 105 EB showed a significantly higher StL/StH ratio with respect to the other groups. 

### 3.3. Number of Vertebrae

Descriptive statistics of the number of vertebrae is shown in Table 8. At both sampling points, the most frequent vertebral formula in abdominal, caudal, and caudal complex regions was, respectively, 9:34:3 (Table 8). Fish displayed an analogous number of vertebrae in every anatomic region among dietary treatments, except the number of caudal vertebrae, which was more variable (Figure S2a), in particular in 31 EA and 31 ED-1 (Table 8). Regarding the total number of vertebrae (Table 8 and Figure S2b), a larger variability was found in 31 EA, 31 ED-1, 105 ED-1, and 105 ED-2 groups.

### 3.4. Vertebral Anomalies

**Figure 2:**
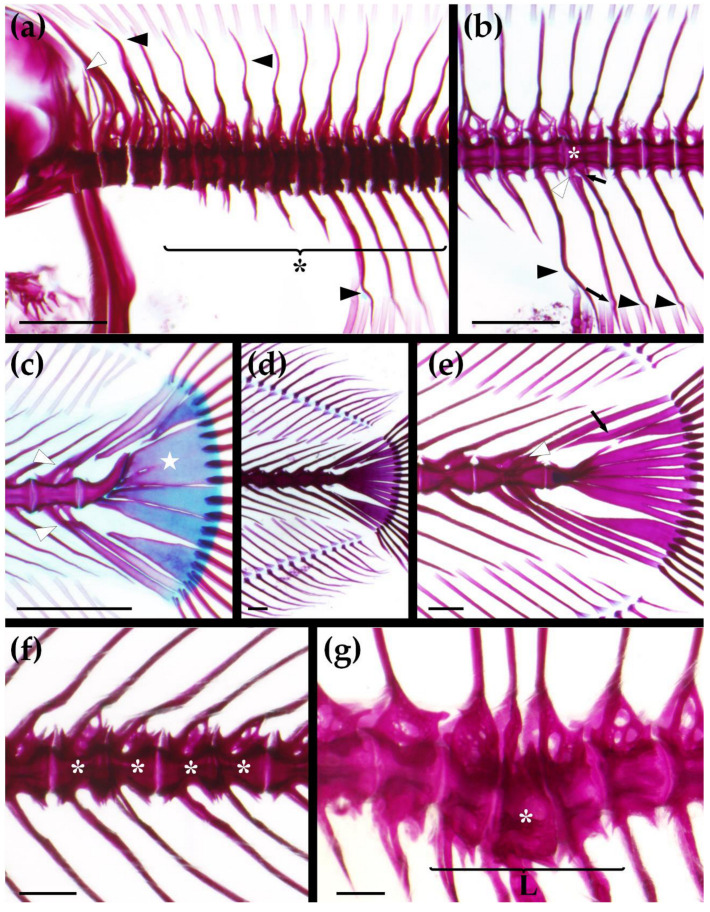
More common anomalies in 31 days after hatching (dah) (**a**–**c**) and 105 dah (**d**–**g**) Senegalese sole (*Solea senegalensis*). Double staining technique with alcian blue and alizarin red. (**a**) Severe deformations affecting both abdominal and caudal vertebrae exhibiting evident flattening of centra and irregularity of vertebral surface and central fusion among the affected elements (asterisk); slight deformities in abdominal neural spines and first caudal haemal spine (black arrowheads); note also the two short neural spines with absence of the neural arch of the first abdominal vertebra (white arrowhead); (**b**) Fusion between the second and third caudal vertebrae with shortening of the first vertebral body (asterisk); incomplete arch (white arrowhead) and alteration of the insertion (short arrow) of the caudal haemal arches; twisted haemal spines (black arrowheads), with a thin layer of bone and slight torsion at the tip of the haemal spine (black arrow); (**c**) Fusion between preurals with fusion of neural and haemal arches (white arrowheads), the union has the appearance of one elongated vertebra; alterations of the shape of epural and hypural 5; fusion among hypurals (white star); (**d**) Overview of the caudal complex in 105 dah Senegalese sole; (**e**) Vertebral fusion between preurals and the last caudal centra, displaying also fusion of preural neural arches (white arrowhead); deformities of hypural 5 and parhypural, denote the thin layer of bony tissue in epural reshaping the element (black arrow); (**f**) Fusions among caudal vertebrae exhibiting an altered orientation of the central structure in opposite directions (asterisks); (**g**) Lordosis in the transition area between abdominal and caudal regions (L); the affected vertebrae are also shorter and deformed, highlights the central vertebra which shows a trapezoidal shape (asterisk). Bars = 500 μm.

Nearly all individuals presented at least one vertebral anomaly (the only exception was one fish from 105 EA; Table 9) and all showed two short neural spines without the neural arch in the anteriormost abdominal vertebra (Figure 2a), which was considered a lot specificity feature and not as an anomaly. Some anomaly typologies listed in Table S2 were not observed in any of the studied specimens, namely fusions and deformations of the parapophysis; bifurcation, number alteration, fusion and incomplete arch in abdominal neural arches and spines; abdominal kyphosis and scoliosis; bifurcation and insertion alteration of the caudal neural arches and spines; insertion alteration of the caudal complex neural arches and spines; bifurcation of the caudal complex haemal arches and spines; bifurcation, number alteration, and insertion alteration of the hypurals, epural, and parhypural (Table 10).

The most common affected skeletal elements were neural/haemal arches and spines as well as caudal complex elements (Table 10). At 31 dah, typologies like twisted caudal haemal spines (Figure 2a,b), fusion and/or bending of hypurals, epural, or parhypural (Figure 2c) were highly recurrent. The caudal complex (Figure 2d) was the most affected region at 105 dah, showing frequent alterations in the shape of caudal complex elements (Figure 2e) and incidences that ranged from 96.0% to 100.0% (Table 10). The frequency of other types of anomalies, for instance, incomplete arches and alterations in the number or insertion of the neural/haemal spines (Figure 2b), was low or absent. Specimens showing VBA and/or VCD ranged from 28% to 48% (Table 9). The most common VBA at 31 dah and 105 dah was the deformation of caudal complex vertebral bodies (20.0–26.0% and 18.0–40.0%, respectively; Table 10), fusions among caudal complex vertebrae (Figure 2c,e), altered shape or shortening (Figure 2a,b) of the vertebral centra were also observed in other regions and sometimes concurrently with vertebral fusions (Figure 2f) or deviations of the rachis (Figure 2g). VCD, namely lordosis (Figure 2f) and scoliosis, were more frequent in the caudal region at 31 dah and in the caudal complex at 105 dah, although with a frequency lower than 12.0% of the individuals of each diet-tank group (Table 10). In addition, some of the anomaly typologies were more frequent or only present in a specific diet-tank group (Table 10). For instance, fish from only two groups (both at 31 dah) showed vertebral fusions in the abdominal region and 31 ED-1 showed the highest prevalence of them. A higher percentage of fish in 31 EA presented deformations of the caudal neural arches and spines and caudal lordosis (31 EA and 105 EA). 105 EB displayed a higher incidence of caudal complex scoliosis, although it affected less than 6% of the specimens. Regarding EC diet, 31 EC-2 and 105 EC groups were almost the only ones to present number and insertion alterations of the caudal complex haemal arches. Moreover, 105 EC showed the highest frequency of caudal complex lordosis. 31 ED-1 and 31 ED-2 showed a higher percentage of fish with the fusion of the caudal haemal arches, and spines and deformations of the caudal complex modified haemal arches and spines. On the other hand, 105 ED-1 and 105 ED-2 groups presented a higher frequency of kyphosis in the caudal complex area.

VBA and/or VCD frequency varied among groups (Table 9). The frequency of individuals with VBA and/or VCD at 105 dah was higher than the correspondent groups at 31 dah, except for 105 EA and 105 ED-1. In the first sampling point, the frequency of these anomalies in 31 EC -1 and 31 EC-2 was the lowest (no more than 30% of affected fish in each tank) compared to other dietary treatments. In turn, at 105 dah, the EA group displayed the lowest percentage of individuals with VBA and/or VCD (28.0%), although, at 31 dah, it was one of the groups with a higher percentage of these anomalies.

**Figure 3:**
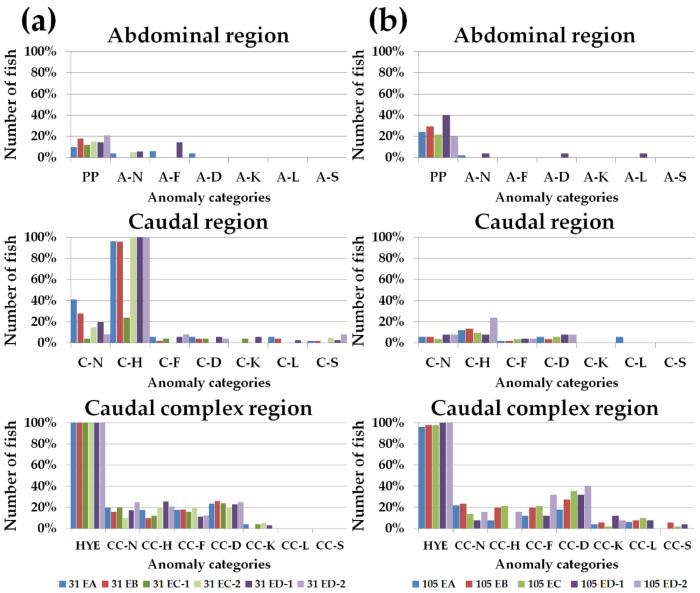
Relative frequency (%) of individuals with at least one anomaly for each group (31 EA, 31 EB, 31 EC-1, 31 EC-2, 31 ED-1, 31 ED-2, 105 EA, 105 EB, 105 EC, 105 ED-1, 105 ED-2) regarding the anatomical regions. (**a**) 31 days after hatching (dah). Haemal caudal elements and the structures of the caudal complex were the most affected; (**b**) 105 dah. The caudal complex region presented the highest frequency of anomalies. A-D: abdominal (A) deformation; A-F: A fusion; A-K: A kyphosis; A-L: A lordosis; A-N: A neural elements; A-S: A scoliosis; CC-D: caudal complex (CC) deformation; CC-F: CC fusion; CC-H: CC haemal elements; CC-K: CC kyphosis; CC-L: CC lordosis; CC-N: CC neural elements; CC-S: CC scoliosis; C-D: caudal (C) deformation; C-F: C fusion; C-H: C haemal elements; C-K: C kyphosis; C-L: C lordosis; C-N: C neural elements; C-S: C scoliosis; HYE: hypurals, epural, parhypural; PP: parapophysis.

The frequencies (percentage) of individuals with at least one anomaly is represented in Figure 3a,b, considering groups and age. These values corresponded to the FM matrix used for the CA analysis. The incidence of each anomaly category varied among groups, although it was not possible to directly visualize a diet-specific pattern. Seventeen of the 40 observed typologies presented an incidence equal or superior to 10.0% in at least one group. These typologies belong to the categories parapophyses (PP), abdominal (A) fusion (A-F), caudal (C) neural elements (C-N), C haemal elements (C-H), caudal complex (CC) neural elements (CC-N), CC haemal elements (CC-H), HYE (hypurals, epural, parhypural), CC fusion (CC-F), CC deformation (CC-D), and CC kyphosis (CC-K) (Table 10 and Figure 3a,b). Some of the predominant anomaly typologies at 31 dah were markedly less common at 105 dah, mostly deformations on caudal neural and haemal elements (C-N and C-H categories, respectively) and fusions among caudal plates (HYE category) in a smaller degree (Table 10 and Figure 3a,b). Alterations in parapophyses (PP) were more frequent in this stage in almost every diet-group. Some anomaly categories appeared only in some dietary groups, although the percentage of affected fish was low (Figure 3a,b). As an example, A deformation (A-D) was only present in fish from groups 31 EA and 105 ED-1, as well as, A lordosis (A-L) was only present in fish from 105 ED-1 group. 31 EA and 105 EA presented also the highest frequency of C lordosis (C-L), while a high percentage of 105 ED-1 and 105 ED-2 groups showed C deformation (C-D) at 105 dah (Figure 3a,b).

**Figure 4:**
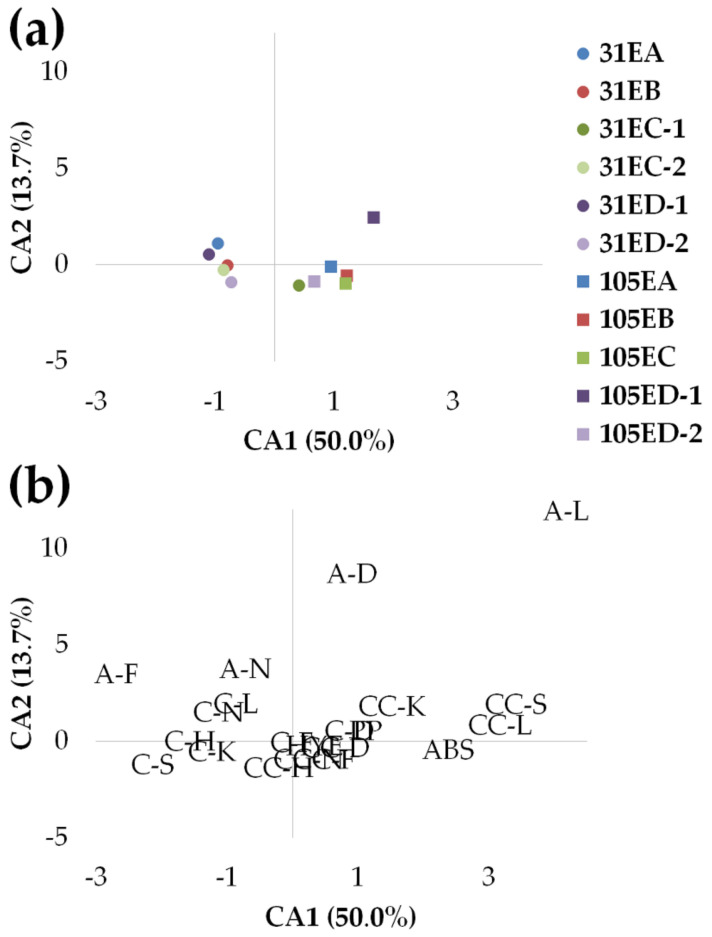
Correspondence analysis results on the frequency matrix (FM) at 31 and 105 days after hatching: ordination model of diet-tank groups (31 EA, 31 EB, 31 EC-1, 31 EC-2, 31 ED-1, 31 ED-2, 105 EA, 105 EB, 105 EC, 105 ED-1, 105 ED-2) (**a**) and descriptor points (**b**) in axis 1 and axis 2. Note the overlapping anomaly categories CC-F, CC-D, CC-N, and HYE (b). A-D: abdominal (A) deformation; A-F: A fusion; A-K: A kyphosis; A-L: A lordosis; A-N: A neural elements; A-S: A scoliosis; ABS: absence of anomalies; CC-D: caudal complex (CC) deformation; CC-F: CC fusion; CC-H: CC haemal elements; CC-K: CC kyphosis; CC-L: CC lordosis; CC-N: CC neural elements; CC-S: CC scoliosis; C-D: caudal (C) deformation; C-F: C fusion; C-H: C haemal elements; C-K: C kyphosis; C-L: C lordosis; C-N: C neural elements; C-S: C scoliosis; HYE: hypurals, epural, parhypural; PP: parapophysis.

Regarding the results obtained from the application of the CA to the FM matrix, the overall variance for the first two correspondence axes (CA1, CA2) was 63.7% (Figure 4a,b). Basically, the ordination model on CA1 and CA2 axes separated the groups on the basis of age: 31 dah groups occupied the negative portion of CA1, whereas fish at 105 dah were located on the positive side of CA1 (Figure 4a). The exception was 31 EC-1, placed in the same quadrant as 105 EC. Moreover, two groups of the ED diet were separated by the CA2 axis: 31 ED-1 and 105 ED-1 located in the positive semiaxis of CA2, and 31 ED-2 and 105 ED-2 in the negative one. The majority of lots were located in the proximity of CA2 origin, but the 105 ED-1 group was the most apart from this axis. It is noteworthy that fish from diets EB and EC were distributed very proximate to each other at both developmental stages, namely 31 EB and 31 EC-2 were located together as well as 105 EB and 105 EC. Regarding the types of anomalies, most of the anomaly categories were located near the axes origins, and some were overlapping (CC-F, CC-D, CC-N, and HYE). A-F, C-L, A neural elements (A-N), and C-N are located in the 2nd quadrant with 31 ED-1 and 31 EA. PP, A-L, A-D, C-D, CC-K, CC scoliosis (CC-S), CC lordosis (CC-L), and ABS plotted in the positive half plane of CA1, together with 105 dah lots and 31 EC-1. Finally, C-H, C kyphosis (C-K), and C scoliosis (C-S) characterized the 31 EB, 31 EC-2, and 31 ED-2 lots.

**Figure 5:**
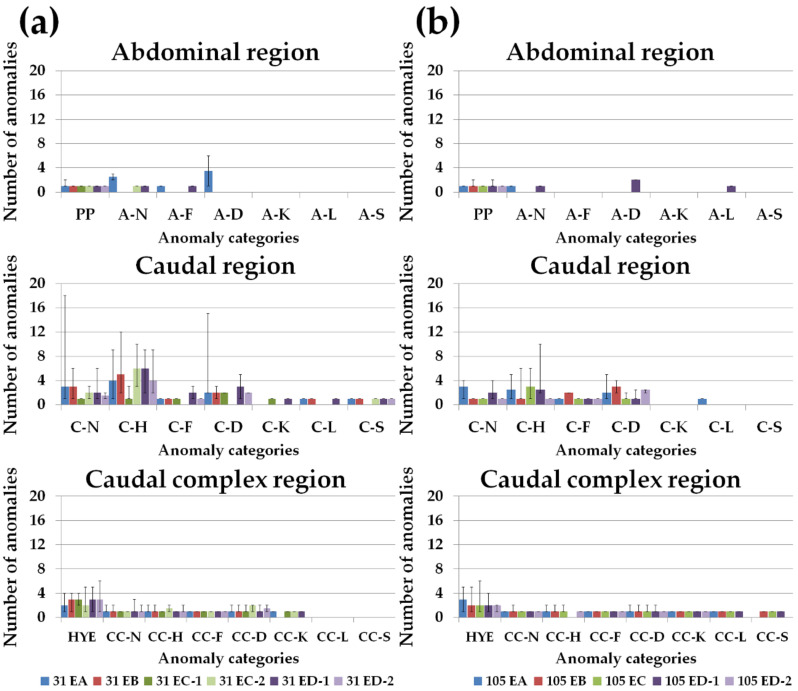
Anomaly index (number of anomalies per malformed specimen) in each group (31 EA, 31 EB, 31 EC-1, 31 EC-2, 31 ED-1, 31 ED-2, 105 EA, 105 EB, 105 EC, 105 ED-1, 105 ED-2). Graphs display median values, maximums, and minimums (**a**) 31 days after hatching (dah). (**b**) 105 dah. A-D: abdominal (A) deformation; A-F: A fusion; A-K: A kyphosis; A-L: A lordosis; A-N: A neural elements; A-S: A scoliosis; CC-D: caudal complex (CC) deformation; CC-F: CC fusion; CC-H: CC haemal elements; CC-K: CC kyphosis; CC-L: CC lordosis; CC-N: CC neural elements; CC-S: CC scoliosis; C-D: caudal (C) deformation; C-F: C fusion; C-H: C haemal elements; C-K: C kyphosis; C-L: C lordosis; C-N: C neural elements; C-S: C scoliosis; HYE: hypurals, epural, parhypural; PP: parapophysis.

Regarding the number of anomalies per specimen (anomaly index), the majority of individuals displayed each anomaly once (Figure 5a,b). The anomalies affecting the haemal arch of caudal vertebrae (C-H) were the ones with the higher recurrent anomalies in all the groups (1-12 anomalous haemal arches in 31 dah and 1-9 in 105 dah affected samples), although one specimen presented 18 altered neural elements at 31 dah (Figure 5a,b). Anomalies affecting hypurals, epural, and parhypural (category HYE) presented a mean charge of at least two anomalies per affected fish in every group (Figure 5a,b). Similar to the frequency of affected individuals, the anomaly index was slightly lower at 105 dah, especially in C-N and C-H (Figure 5b), but no clear dietary trend was detectable in both sampling points.

## 4. Discussion

The results of the nutritional composition analysis showed that the efficiency of enrichment of metanauplii was uncorrelated with the values reported in the manufacture’s label for Vit. C, D_3_, total n-3 PUFA, EPA but not for P and DHA levels. This can be due to the lack of information for some of the nutrients on the label of commercial product (especially EB enrichment), preventing a more complete comparison. However, the metanauplii administered to the different experimental groups of postlarvae showed different levels of the analyzed nutrients. In particular, EB metanauplii had the highest level of Vit. C, Vit. D_3_, and total n-6 PUFA, EC the highest levels in P and DHA, ED total n-3 PUFA and EPA, whilst the EA metanauplii the highest level of (n-3)/(n-6) PUFA ratio. There is scarce information on the nutritional requirements for Senegalese sole larvae and postlarvae. In the study of Boglino et al. [4], the recommended absolute nutritional levels for *Artemia* metanauplii were 9.5% and 3.1% TFA, for DHA and EPA, respectively and 5.2 and 3.0 for (n−3)/(n−6) PUFA and DHA/EPA, as more suitable for Senegalese sole larval development [4]. In this study, the DHA and DHA/EPA levels by one hand and EPA values by another hand were considerably lower and higher, respectively, than the recommended ones. However, (n−3)/(n−6) PUFA requirements were fulfilled in EA and ED enrichments, although the total amount of (n-3) PUFA and (n-6) PUFA were below the values of the reference diet used for the nutritional recommendations in Boglino et al. [4] study.

Enriched *Artemia* metanauplii constituted the unique food item offered to the larvae from the 5th up to the 30th dah: so far, data obtained from analyses carried out on 31 dah soles describe the short-time effect of the enrichments. From the 31st dah onward, the gradual integration of the artificial feed was applied. The values for the nutritional composition in Senegalese soles sampled at 29 dah variated among diets and replicates. Discrepancies between replicates were especially observed in EA-1 and EA-2 fish in total n-3 HUFA and Vit. C (to a lesser extent) levels. Still, fatty acid content in Senegalese sole regarding (n-3) PUFA, (n-6) PUFA, DHA, and the ratio DHA/EPA were inferior to those reported in Boglino et al. [4] for fish fed the recommended diet at similar rearing stages. Comparing the composition of the metanauplii with the sampled fish, the impact of certain dietary nutrients was noticeable in the postlarvae composition. In particular, Vit. D_3_ and total n-6 PUFA levels were prominently higher in EB metanauplii and, consistently, both groups of fish EB-1 and EB-2 showed also higher levels of this vitamin and fatty acids. However, differences were also found between metanauplii and fish composition, namely, in the values of Vit. C, Ca, P, Ca/P, and (n-3)/(n-6), where the highest levels belonged to EB, ED, EC, ED, EA metanuplii, respectively, and were not higher in the corresponding fish groups. The basic biochemical composition of metanauplii and postlarvae was used for guidance only, and more exhaustive analysis should be performed to evaluate the significance of the differences observed.

Until 31 dah, the survival rate remained comparable with other studies in Senegalese sole [4,31], and no significant differences were found among the experimental groups. However, at 105 dah, 105 EA and 105 ED-1 groups showed the significant highest and lowest survival rates, respectively. To our knowledge, the effect of enrichment products at early juvenile stages (105 dah) has not been studied on this species and survival data are scarce. However, since 105 ED-1 and 105 ED-2 showed significant intra-replicate differences, the hypothetical effect of the diet on the survival rate could be discarded in these groups.

Regarding morphometrics, the soles reared at the lowest densities (EC-2: 84 postlarvae/L and ED-1: 69 postlarvae/L) showed the significant highest values of StL, StH, and StL/StH. In particular, both StL and StH median values recorded in the replicate 31 EC-2 and 31 ED-1 resulted significantly higher than those reared at intermediate densities (31 EB and 31 ED-2: ~95 postlarvae/L, at 15 dah). Conversely, 31 EA maintained at the highest density (103–108 postlarvae/L) showed intermediate (and not the lowest) biometric values.

Consequently, it can be hypothesized that the presence of significant intra-replicate and some inter-group differences in lengths observed at 31 dah and in survival rates at 105 dah could be imputed to differences in the densities (number of postlarvae/L) during the experimental rearing. Lower stocking densities seem to induce faster growth in Senegalese sole in the first rearing phase. In the same sense, studies in Senegalese sole observed that juveniles under high density tended to grow less than fish at low density [32,33]. This effect is surely detectable up to 31 dah in this experiment but it seems to be diluted later on, as it was not detectable at 105 dah. Accordingly, Salas-Leiton et al. [32] detected differences in growth among density groups at initial phases but not at the end, suggesting a compensatory growth. Certainly, these comparisons should be carefully interpreted since in this study, the stocking density was measured as the number of postlarvae/L, instead of using a biomass/volume, biomass/bottom surface or fish surface/bottom surface criterions as in the literature cited above or in certain stages of this species farming practices [19]. The observation that the biometric data was not significantly different between dietary groups at 105 dah suggests that the enrichments did not exercise any detectable effects on length and height of Senegalese sole at this age. However, Senegalese sole from 105 EA and 105 EB presented a slenderer profile than other groups, as shown by the significantly higher ratio StL/StH. In this sense, at an industrial level, the elongated external profile of the sole is preferred than a “plaice-like” form (Riaza personal communication) and, therefore, a valuable feature for the market.

Under these experimental conditions, most fish from all groups presented 9:34:3 vertebrae in abdominal, caudal, and caudal complex regions, respectively. These meristic counts agree to those reported by Gavaia et al. [2] in reared Senegalese sole. However, 31 EA and 31 ED-1 showed variation in the minimum number of caudal vertebrae and in the percentage of individuals showing the median values (%Im), compared with the other groups at 31 dah but not at 105 dah. Given that the number of vertebrae is established during embryogenesis, and the genetic tests demonstrated that the larvae used for all the replicates and diet groups came from a single female and male, these variations could be imputed only to the presence of completely fused caudal vertebrae, counted as one vertebra. However, in 31 EA and 31 ED-1 specimens, no complete fusions were observed in the caudal region.

Anomalies of the vertebral column were very common, reaching 99.7% of the specimens. This value is consistent with other reports in this species where the frequency of deformed individuals reached 35–44% [2,34], 70–80% [4,5,35], and up to 100% in some cases [3,6]. In contrast, in wild fish, studies observed 19% of malformations [5]. In all groups, VBA and/or VCD frequencies exceeded the 20% reference value for severely deformed fish in intensive production [8], a relevant and sensible value in the industry [36]. Some anomaly categories were very frequent in all groups, indicating the presence of a common trend or mainstay of vertebral deformities. The elevated incidence of anomalies in the caudal complex structures is comparable with other reports for this species [3,5,6,7] and was repeatedly common in all diet and age groups. In this study, the absence of neural/haemal vertebral arches in contrast with supernumerary elements was indistinctly considered in the same anomaly category, although infrequent. Further studies analyzing these events separately (as in Martini et al. [37] and Ferreri et al. [38]) could help to understand the pathway triggered by distinct factors on the development of such different anomalies.

The incidence of anomaly categories showed some variations regarding dietary treatments. In particular, only 31 EA and 105 EA showed A-D and C-L and completely normal specimens (ABS) at 31 and 105 dah (respectively). 105 ED-1 displayed a higher number of individuals with A-D and A-L, although these percentages were below 10%. At 31 dah, VBA and/or VCD frequency was one of the highest in 31 EA, while, 105 EA displayed the lowest percentage of individuals with these anomalies. Therefore, the hypothetical association of some anomalies with one diet at 31 dah was not maintained at 105 dah, and a clear effect of the diets on skeletogenesis was not observed. Other studies in Senegalese sole did not find a clear influence of *Artemia* spp. enrichment products on the incidence of the overall skeletal anomalies [4,31]. However, fish fed certain dietary enrichment products showed differences regarding the ossification degree, the frequency of vertebral fusions, and deformities of the caudal complex structures, especially the modified neural and haemal spines [4]. That such a result was not observed in the present study may be due to the use of alternative commercial enrichments, or due to different sample/replicate size and rearing protocols. Moreover, the existence of common anomalies to all diets in both stages may indicate that other causes could have contributed to the onset of such alterations. Skeletal malformations are a multi-factorial problem related to several causes, mainly environmental and nutritional factors [8,9]. Environmental agents affecting Senegalese sole skeleton include the temperature, hypercapnia, photoperiod, and the exposure of high amounts of triclosan [39,40,41,42]. In other reared marine species, i.e., gilthead seabream *Sparus aurata*, Linnaeus [27], and in zebrafish *Danio rerio,* Hamilton [37], the rearing densities influence the types and frequency of skeletal anomalies. Thus, the unexpected density differences observed in this study could be affecting the skeletal anomaly profile of Senegalese sole. However, the presence of highly frequent types of anomalies in all groups may indicate the unlikelihood of a single effect of rearing density. Moreover, it could be possible that the effects of rearing density and enrichment products are inconspicuous due to other hypothetical causative factors of the common mainstay of anomalies. Further research is required in order to assess the role of rearing conditions, in particular of the stocking density, over the development of skeletal deformities in Senegalese sole.

Concurrently, CA results ordered fish according to the age groups (31 and 105 dah) in separate CA1 semiaxes, establishing different anomaly patterns for each age. The only exception to this was 31 EC-1, which was located in the same quadrant as 105 EB, 105 EC, and 105 ED-2. At 105 dah, there was a reduction in the number of fish displaying certain anomalies as A-N, A-F, C-K, C-L, C-S, but markedly C-N and C-H. The substantial diminution of alterations of caudal neural and haemal elements might be due to an adaptive response. Teleost skeleton can show considerable plasticity and adaptive response to loading conditions [43,44,45]. Fish bone has the ability to remodel and model some vertebral fusions and repair fractures [46,47,48]. Recently, studies in gilthead seabream and Atlantic salmon (*Salmo salar,* Linnaeus) reported even the possibility of recovery of severe skeletal anomalies [49,50]. In this sense, the presence of a thin layer of bony tissue in some deformed structures at both stages could indicate bone formation, helping to strengthen the concave side of the spines or plates. These altered elements might be undergoing remodeling and modeling processes and perhaps afterward acquire a non-deformed shape, leading to the attenuation of the incidence of spine deformities at later stages [51]. Moreover, EB, EC, and ED diets presented a higher frequency of individuals with VBA and/or VCD at 105 dah than the respective groups at 31 dah. These variations in the profile of the abnormalities with age might be due to certain nutrients acting later in development or predispose sole to vertebral deformities, as suggested for Atlantic salmon juveniles fed with inadequate phosphorus diet [52]. Further investigation is needed to deepen the histopathologic features of Senegalese sole’s bone adaptive processes.

Globally, in the first sampling point, 31 EC-1 fish showed slightly better performances in terms of larval quality, with lower incidences related to VBA and VCD and deformities in haemal elements. Moreover, CA output located this group among 105 dah groups, indicating a relatively similar anomaly pattern in these fish. Considering the nutritional composition of Senegalese sole postlarvae, EC-1 showed intermediate values of all analyzed nutrients. Nutritional composition of *Artemia* metanauplii enriched with EC displayed elevated DHA content and ratio DHA/EPA (2.52% TFA, 0.35, respectively), although greatly diminished compared to the recommended dietary levels [4]. In turn, DHA and the ratio DHA/EPA dietary amounts were reduced in 40 dah Senegalese sole, in comparison to the present study. In other fish species, low DHA diets may influence early mineralization and the incidence of skeletal deformities [53,54]. In the present study, the discrepancies on the anomaly frequency between 31 EC-1 and 31 EC-2 fish (both fed EC metanauplii) prevented the detection of a clear role of the DHA dietary content on skeletogenesis. Considering the overall quality of 105 dah juveniles, EA diet presented lower incidences of VBA and/or VCD and deformations of the caudal complex elements. The nutritional composition of Senegalese sole fed with EA-enriched metanauplii stands out a reduced phosphorous content resulting in a higher ratio Ca/P respect to the other groups, whereas EA metanauplii showed an unexpected intermediate/high P content. However, studies in rainbow trout (*Oncorhynchus mykiss,* Walbaum) showed that the whole body phosphorous concentration responded to its dietary intake [13]. In Atlantic salmon, inadequate phosphorus content in the diet at the juvenile stage could predispose to development of compressed vertebral bodies as postsmolts [52]. Nevertheless, the primary phosphorous deficiency should not be considered the only cause of bone disorders observed in *Salmo salar* since the resulting defect in mineralization was not consistent with the appearance of skeletal anomalies [55,56]. Reports in Senegalese sole studied the role of vitamin A [3,57] and K [15] as well as arachidonic acid [34] on skeletal deformities. To optimize sole’s nutrition, it is also important to consider the balance and interactions among nutrients [4] as well as the changes in the nutritional profile of live preys that may occur depending on culture/enrichment conditions [18] or certain *Artemia* spp. metabolic pathways [4]. Therefore, with the present data, it is greatly difficult to detach a single enriching nutrient that could be having an effect on the development of vertebral anomalies.

## 5. Conclusions

In conclusion, this study carried out to describe the “long-term” effects of *Artemia*‘ enrichment administered during larval stages seems to confirm what reported by Boglino et al. [4]: no clear influences of *Artemia* enrichment is detectable on the overall frequency of skeletal deformities in reared Senegalese sole postlarvae and juveniles. A high percentage of individuals exhibited skeletal anomalies in every dietary group. The main anomalies consisted of alterations of neural/haemal elements, as well as caudal complex plates. Regarding VBA and VCD overall incidence, 31 EC-1 and 105 EA groups showed better performance at 31 and 105 dah, respectively. CA results indicated a different anomaly pattern among stages. The existence of very frequent anomalies points to the presence of a common mainstay of vertebral deformities unrelated to the type of enrichment, and the influence of other factors like rearing conditions on the development and progression of such alterations. This study, however, detected a possible effect of the rearing densities on the fish growth and on the skeletal anomaly onset, as already described in other marine reared species. These results imply considerations to be done by farmers on the economic advantages deriving from the optimization of rearing conditions vs the investment on special *Artemia* enrichments.

## Figures and Tables

**Table 1:** Number of individuals in each replicate tank (EA-1, EA-2, EB-1, EB-2, EC-1, EC-2, ED-1, ED-2) at 15 days after hatching. Different numbers (with the same prefix) refer to each replicate from the same enrichment product.

Dietary Replicates	EA-1	EA-2	EB-1	EB-2	EC-1	EC-2	ED-1	ED-2
Number of individuals	2705	2568	2367	2354	2349	2108	1728	2366

**Table 2:** Feeding regime during the experiment. Rotifers (*Brachionus plicatilis*) were enriched with microalgae (*Isochrysis galbana*). *Artemia* spp. metanauplii were enriched with four different commercial products (EA, EB, EC, ED). Dah: days after hatching.

Age (dah)	Diet	Feeding Density	Times/Day
2–4	Enriched rotifers	6**–**7 rotifers mL^−1^	2
5–8	Enriched rotifers	2**–**3 rotifers mL^−1^	2
	*Artemia* spp. nauplii	1**–**3 nauplii per 10 mL	2
8–14	Enriched *Artemia* spp metanauplii	6**–**24 metanauplii per 10 mL	2
15–30	Enriched *Artemia* spp metanauplii	40**–**80 metanauplii mL^−1^	4
31–38	Commercial weaning diet		10**–**20
38–105	Commercial inert diet		

**Table 3:** Nutritional information for each enrichment (EA, EB, EC, ED) according to the manufacture’s label. DHA: docosahexaenoic acid; dw: dry weight; EPA: eicosapentaenoic acid; HUFA: highly unsaturated fatty acids; uns: unspecified.

Nutritional Analysis	EA	EB	EC	ED
Vitamin A (mg kg^−1^ dw)	45	uns	43	47
Vitamin C (mg kg^−1^ dw)	33,000	uns	38,571	21,053
Vitamin D_3_ (µg kg^−1^ dw)	1250	uns	714	395
Phosphorous (g kg^−1^ dw)	uns	uns	28,571	6316
Total n-3 HUFA (mg g^−1^ dw)	150	105	186	150
20:5n-3 (EPA) (mg g^−1^ dw)	10	uns	14	uns
22:6n-3 (DHA) (mg g^−1^ dw)	130	uns	157	uns
DHA/EPA	13	4	11	9

**Table 4:** Nutritional composition of *Artemia* spp. metanauplii enriched with four different products (EA, EB, EC, ED). DHA: docosahexaenoic acid; EPA: eicosapentaenoic acid; PUFA: polyunsaturated fatty acids; % TFA: percentage of the total fatty acids; ww: wet weight.

Nutritional Analysis	EA	EB	EC	ED
Vitamin A (mg kg^−1^ *ww*)	*	*	*	*
Vitamin C (mg kg^−1^ *ww*)	957	1113	1007	1066
Vitamin D_3_ (µg kg^−1^ *ww*)	15	28	10	12
Calcium (Ca) (g kg^−1^ *ww*)	0.195	0.155	0.141	0.229
Phosphorous (P) (g kg^−1^ *ww*)	1.085	0.879	1.154	0.980
Ratio Ca/P	0.18	0.18	0.12	0.23
Total n-3 PUFA (% TFA)	34.34	32.98	30.32	34.96
Total n-6 PUFA (% TFA)	5.87	8.97	6.83	6.70
20:5n-3 (EPA) (% TFA)	8.09	5.85	7.17	8.17
22:6n-3 (DHA) (% TFA)	1.81	1.82	2.52	1.91
DHA/EPA	0.22	0.31	0.35	0.23
(*n*-3)/(*n*-6)	5.85	3.68	4.44	5.22

* Asterisks represent values below the equipment detection limit (<0.5).

**Table 5:** Basic nutritional composition of Senegalese sole (*Solea senegalensis*) at 29 days after hatching for each tank (EA-1, EA-2, EB-1, EB-2, EC-1, EC-2, ED-1, ED-2). DHA: docosahexaenoic acid; EPA: eicosapentaenoic acid; PUFA: polyunsaturated fatty acids; % TFA: percentage of the total fatty acids; *ww*: wet weight.

Nutritional Analysis	EA-1	EA-2	EB-1	EB-2	EC-1	EC-2	ED-1	ED-2
Vitamin A (mg kg^−1^ *ww*)	0.7	0.6	0.6	0.7	0.8	0.7	0.9	1
Vitamin C (mg kg^−1^ *ww*)	323	272	278	270	320	325	316	332
Vitamin D_3_ (µg kg^−1^ *ww*)	5	6	13	14	6	*	*	*
Calcium (Ca) (g kg^−1^ *ww*)	5.502	6.309	6.000	5.752	5.472	5.370	5.057	5.169
Phosphorous (P) (g kg^−1^ *ww*)	3.069	2.415	4.939	4.627	4.435	4.415	4.183	4.315
Ratio Ca/P	1.79	2.61	1.21	1.24	1.23	1.22	1.21	1.20
Total n-3 PUFA (% TFA)	22.10	28.83	23.44	26.42	25.49	28.05	26.02	31.79
Total n-6 PUFA (% TFA)	8.49	7.81	9.76	10.05	8.35	9.40	6.70	7.73
20:5n-3 (EPA) (% TFA)	2.23	2.38	3.13	2.98	2.46	3.34	3.07	3.05
22:6n-3 (DHA) (% TFA)	9.22	7.23	9.29	5.88	9.05	12.96	9.00	8.36
DHA/EPA	4.13	3.04	2.97	1.97	3.68	3.88	2.93	2.74
(*n*-3)/(*n*-6)	2.60	3.69	2.40	2.63	3.05	2.98	3.88	4.11

* Asterisks represent values below the equipment detection limit (<5).

**Table 6:** Postlarvae diet-tank groups considered for survival, measurements, and skeletal evaluation. dah: days after hatching.

Postlarvae Groups	Age	Enrichment	Replicate
31 EA	31 dah	EA	EA-1 + EA-2
31 EB	31 dah	EB	EB-1 + EB-2
31 EC-1	31 dah	EC	EC-1
31 EC-2	31 dah	EC	EC-2
31 ED-1	31 dah	ED	ED-1
31 ED-2	31 dah	ED	ED-2
105 EA	105 dah	EA	EA-1 + EA-2
105 EB	105 dah	EB	EB-1 + EB-2
105 EC	105 dah	EC	EC-1 + EC-2
105 ED-1	105 dah	ED	ED-1
105 ED-2	105 dah	ED	ED-2

**Table 7:** Daily survival rate (%) and biometric results for standard length (StL), standard height (StH), and the ratio StL/StH for each diet-tank group (31 EA, 31 EB, 31 EC-1, 31 EC-2, 31 ED-1, 31 ED-2, 105 EA, 105 EB, 105 EC, 105 ED-1, 105 ED-2): mean ± standard error of the mean, median, minimum, and maximum. dah: days after hatching; SEM: standard error of the mean; Surv.: daily survival rate.

Sampling Points		31 dah						105 dah				
Diet-Tank Groups		31 EA	31 EB	31 EC-1	31 EC-2	31 ED-1	31 ED-2	105 EA	105 EB	105 EC	105 ED-1	105 ED-2
Surv. (%)	Mean ± SEM	96.7 ± 1.2	96.3 ± 1.4	95.6 ± 1.4	95.0 ± 1.6	94.9 ± 1.9	96.0 ± 1.4	82.9 ± 0.7	80.5 ± 0.9	80.3 ± 0.8	74.2 ± 1.1	81.1 ± 0.8
	Median	98.9	98.7	98.0	97.6	98.1	98.4	79.9 ^a^	77.2 ^b^	77.2 ^b^	69.3 ^c^	77.8 ^b^
	Minimum	84.9	83.4	82.7	80.6	77.4	83.2	76.2	72.2	72.1	64.6	74.3
	Maximum	100.0	100.0	100.0	100.0	100.0	100.0	100.0	100.0	100.0	100.0	100.0
StL (cm)	Mean ± SEM	1.692 ± 0.020	1.670 ± 0.017	1.677 ± 0.026	1.802 ± 0.023	1.768 ± 0.022	1.662 ± 0.024	3.9 ± 0.133	3.7 ± 0.109	3.9 ± 0.100	3.9 ± 0.146	3.7 ± 0.151
	Median	1.72 ^b,c^	1.66^c^	1.69 ^b,c^	1.80 ^a^	1.79 ^a,b^	1.63 ^c^	3.8	3.6	3.9	3.9	3.6
	Minimum	1.10	1.25	1.28	1.63	1.31	1.47	2.5	2.3	2.8	2.7	2.5
	Maximum	1.89	1.93	1.94	1.98	2.01	1.93	6.7	5.9	6.4	5.8	5.5
StH (cm)	Mean ± SEM	0.583 ± 0.006	0.575 ± 0.006	0.581 ± 0.009	0.619 ± 0.008	0.607 ± 0.008	0.578 ± 0.009	1.2 ± 0.047	1.2 ± 0.038	1.3 ± 0.037	1.3 ± 0.058	1.2 ± 0.050
	Median	0.59 ^a,b^	0.57 ^b^	0.59 ^a,b^	0.61 ^a^	0.61 ^a^	0.57^b^	1.2	1.1	1.2	1.3	1.1
	Minimum	0.43	0.44	0.44	0.57	0.44	0.50	0.7	0.8	0.8	0.8	0.8
	Maximum	0.65	0.67	0.67	0.68	0.70	0.67	2.1	2.0	2.1	2.1	1.8
StL/StH	Mean ± SEM	2.902 ± 0.012	2.906 ± 0.009	2.888 ± 0.015	2.913 ± 0.013	2.915 ± 0.012	2.883 ± 0.015	3.3 ± 0.028	3.2 ± 0.024	3.1 ± 0.025	3.0 ± 0.033	3.1 ± 0.030
	Median	2.9	2.9	2.9	2.9	2.9	2.9	3.2 ^a^	3.2 ^a,b^	3.1 ^b^	3.0 ^c^	3.1 ^b,c^
	Minimum	2.6	2.8	2.7	2.8	2.8	2.7	2.8	2.9	2.8	2.6	2.7
	Maximum	3.0	3.1	3.0	3.0	3.1	3.0	3.7	3.7	3.6	3.4	3.5

^1^ Medians with a different superscript letter denote significant differences in the distribution among dietary-tank groups for each sampling point (Kruskall-Wallis test, *p* < 0.05).

**Table 8:** Number of vertebrae in abdominal, caudal, and caudal complex regions for each diet-tank group (31 EA, 31 EB, 31 EC-1, 31 EC-2, 31 ED-1, 31 ED-2, 105 EA, 105 EB, 105 EC, 105 ED-1, 105 ED-2): median, minimum, and maximum values and occurrences of individuals showing the median values (%Im). dah: days after hatching.

Sampling Points		31 dah						105 dah				
Meristic Counts		31 EA	31 EB	31 EC-1	31 EC-2	31 ED-1	31 ED-2	105 EA	105 EB	105 EC	105 ED-1	105 ED-2
Abdominal Vertebrae	Median	9	9	9	9	9	9	9	9	9	9	9
	Minimum	8	8	9	8	8	9	8	8	8	8	8
	Maximum	9	9	9	9	9	9	9	9	9	9	9
	%Im	98.0	98.0	100.0	95.0	97.1	100.0	98.0	98.0	98.0	92.0	96.0
Caudal Vertebrae	Median	34	34	34	34	34	34	34	34	34	34	34
	Minimum	32	33	33	33	33	33	33	33	33	33	33
	Maximum	35	35	34	35	35	35	35	35	35	35	35
	%Im	56.9	88.0	88.0	85.0	65.7	83.3	86.0	88.2	88.2	72.0	84.0
Caudal Complex Vertebrae	Median	3	3	3	3	3	3	3	3	3	3	3
	Minimum	2	2	2	3	2	2	2	2	2	2	2
	Maximum	3	3	3	3	4	4	3	3	3	3	3
	%Im	90.2	90.0	88.0	100.0	88.6	91.7	88.0	90.2	84.3	88.0	80.0
Total Vertebrae	Median	46	46	46	46	46	46	46	46	46	46	46
	Minimum	44	45	45	45	45	45	44	45	45	45	44
	Maximum	47	47	46	46	47	47	46	46	46	46	46
	%Im	58.8	76.0	76.0	90.0	68.6	91.7	82.0	80.4	74.5	68.0	72.0

**Table 9:** General results on the incidence of skeletal anomalies for each group (31 EA, 31 EB, 31 EC-1, 31 EC-2, 31 ED-1, 31 ED-2, 105 EA, 105 EB, 105 EC, 105 ED-1, 105 ED-2): frequency of individuals displaying at least one anomaly (%); frequency of skeletal abnormalities with respect to the total number of anomalies observed in each age group; frequency of individuals with vertebral body anomalies (VBA) and/or vertebral column deviations (VCD) (%); percentage of VBA and/or VCD in the total number of anomalies of that diet-tank-age group.

Sampling Points	31 dah						105 dah				
	31 EA	31 EB	31 EC-1	31 EC-2	31 ED-1	31 ED-2	105 EA	105 EB	105 EC	105 ED-1	105 ED-2
**Frequency of individuals with at least one anomaly (%)**	100.0	100.0	100.0	100.0	100.0	100.0	98.0	100.0	100.0	100.0	100.0
**Frequency of anomalies (%)**	26.6	24.3	5.6	10.8	20.6	12.0	23.8	27.4	24.5	11.1	13.2
**Frequency of individuals with VBA and/or VCD (%)**	41.2	32.0	28.0	30.0	42.9	33.3	28.0	39.2	41.2	40.0	48.0
**Frequency of VBA and/or VCD (%)**	12.3	7.1	15.1	6.3	8.7	7.9	16.4	18.0	22.5	24.7	24.3

**Table 10:** Relative frequency (%) of affected individuals by each anomaly type (categories code name is between parenthesis) for each group (31 EA, 31 EB, 31 EC-1, 31 EC-2, 31 ED-1, 31 ED-2, 105 EA, 105 EB, 105 EC, 105 ED-1, 105 ED-2). Empty cells denote a frequency equal to zero (0). Bold values denote some typologies more frequent or predominantly present in a specific diet-tank group. dah: days after hatching; VBA: vertebral body anomalies; VCD: vertebral column deviations.

Region	Skeletal Elements	Code	Anomalies Typology	31 dah						105 dah				
				31 EA	31 EB	31 EC-1	31 EC-2	31 ED-1	31 ED-2	105 EA	105 EB	105 EC	105 ED-1	105 ED-2
Abdominal region	Parapophysis	(PP)	Bifurcation	2.0						2.0				
			Number alteration	9.8	18.0	12.0	15.0	14.3	20.8	22.0	27.5	21.6	40.0	20.0
			Insertion alteration								11.8		12.0	8.0
	Neural arches and spines	(A-N)	Number alteration							2.0				
			Deformation	3.9			5.0	5.7					4.0	
	VBA	(A-F)	Fusion	5.9				**14.3**						
		(A-D)	Deformation	3.9									4.0	
	VCD	(A-L)	Lordosis										4.0	
Caudal region	Neural arches and spines	(C-N)	Number alteration	2.0										
			Fusion	2.0				2.9		2.0				8.0
			Incomplete arch	2.0			5.0			2.0	2.0			
			Deformation	**39.2**	28.0	4.0	10.0	17.1	8.3	6.0	3.9	3.9	8.0	
	Haemal arches and spines	(C-H)	Bifurcation	2.0	2.0									4.0
			Number alteration								2.0		4.0	4.0
			Insertion alteration	2.0	4.0	12.0		8.6	12.5	4.0		3.9		4.0
			Fusion					**2.9**	**8.3**		2.0			**4.0**
			Incomplete arch	7.8	6.0	4.0		5.7	12.5	2.0			4.0	4.0
			Deformation	94.1	96.0	16.0	100	100	100	10.0	9.8	5.9	4.0	12.0
	VBA	(C-F)	Fusion	5.9	2.0	4.0		5.7	8.3	2.0	2.0	3.9	4.0	4.0
		(C-D)	Deformation	5.9	4.0	4.0		5.7	4.2	6.0	3.9	5.9	8.0	8.0
	VCD	(C-K)	Kyphosis			4.0		5.7						
		(C-L)	Lordosis	**5.9**	4.0			2.9		**6.0**				
		(C-S)	Scoliosis	2.0	2.0		5.0	2.9	8.3					
Caudal complex	Neural arches and spines	(CC-N)	Bifurcation											4.0
			Number alteration	9.8	4.0	12.0		8.6	4.2	2.0	7.8	2.0	8.0	
			Fusion	7.8	10.0	4.0	10.0	2.9	12.5	10.0	7.8	9.8		8.0
			Incomplete arch	2.0	2.0									
			Deformation	3.9	2.0	4.0		8.6	12.5	10.0	7.8	2.0		4.0
	Haemal arches and spines	(CC-H)	Number alteration	2.0			**5.0**				2.0	**3.9**		
			Insertion alteration				**5.0**				2.0	**5.9**		
			Fusion	9.8	6.0	4.0	10.0	11.4	8.3	6.0	9.8	7.8		12.0
			Incomplete arch	5.9	4.0				8.3			2.0		
			Deformation	5.9	2.0	8.0	5.0	**14.3**	**8.3**	2.0	7.8	5.9		4.0
	Hypurals, epural, parhypural	(HYE)	Fusion	84.3	88.0	88.0	85.0	94.3	95.8	30.0	25.5	11.8	4.0	20.0
			Deformation	90.2	92.0	100.0	90.0	91.4	100.0	96.0	96.1	96.1	100.0	100.0
	VBA	(CC-F)	Fusion	17.6	18.0	16.0	20.0	11.4	12.5	12.0	19.6	21.6	12.0	32.0
		(CC-D)	Deformation	23.5	26.0	24.0	20.0	22.9	25.0	18.0	27.4	35.3	32.0	40.0
	VCD	(CC-K)	Kyphosis	3.9		4.0	5.0	2.9		4.0	5.9	2.0	**12.0**	**8.0**
		(CC-L)	Lordosis							6.0	7.8	**9.8**	8.0	
		(CC-S)	Scoliosis								**5.9**	2.0	4.0	

Bold values denote some typologies more frequent or predominantly present in a specific diet-tank group.

## Data Availability

Data available on request due to restrictions eg., privacy or ethical. The data presented in this study are available on request from the corresponding author. The data are not publicly available due to confidentiality with the funding company.

## Supplementary Materials

The following are available online at https://www.mdpi.com/2076-2615/11/1/22/s1, Figure S1: Scheme illustrating the annotation method for fish groups. Figure S2: Box and whiskers plots regarding the number of caudal vertebrae and total number of vertebrae for each group. Table S1: Number of sampled specimens of each replicate tank at 31 and 105 days after hatching for meristic counts and skeletal anomalies detection. Table S2: Typology of skeletal anomalies that were considered in this study for each anatomical region. Table S3: Fish density (number of fish/L) for each replicate tank at different points of the experiment. Table S4: Daily survival rate (%) from 15 to 105 days after hatching (dah) for each replicate tank.
