# Supplementary material for: Skeletal Anomalies in Senegalese Sole (Solea senegalensis, Kaup) Fed with Different Commercial Enriched Artemia: A Study in Postlarvae and Juveniles
Source: Animals (Basel). 2020 Dec 24;11(1):22. doi: 10.3390/ani11010022 (PMC7823604; doi:10.3390/ani11010022)
Supplement: Supplementary file 1 [file animals-11-00022-s001.pdf]

animals

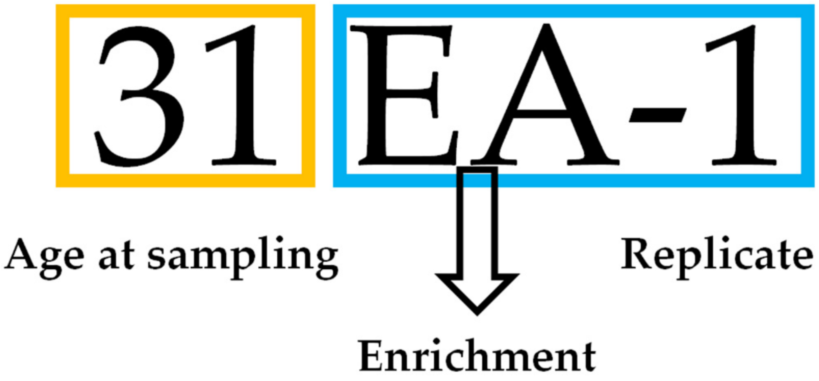

Figure S1. Scheme illustrating the annotation method for fish groups. The age, enrichment product and tank replicate were integrated in the same label.

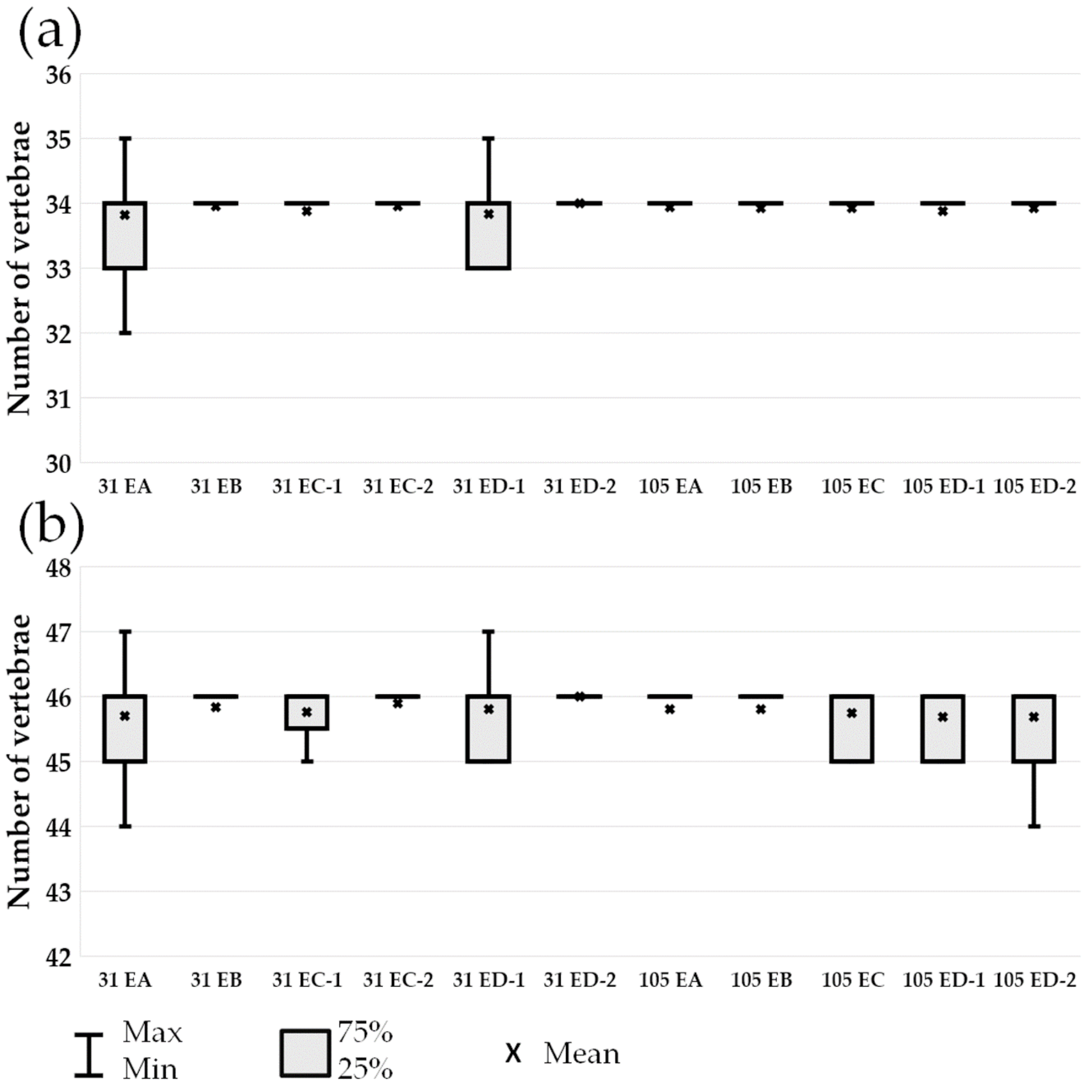

Figure S2. Box and whiskers plots regarding the number of caudal vertebrae (**a**) and total number of vertebrae (**b**) for each group (31 EA, 31 EB, 31 EC-1, 31 EC-2, 31 ED-1, 31 ED-2, 105 EA, 105 EB, 105 EC, 105 ED-1, 105 ED-2).

Table S1. Number of sampled specimens of each replicate tank (EA-1, EA-2, EB-1, EB-2, EC-1, EC-2, ED-1, ED-2) at 31 and 105 days after hatching for meristic counts and skeletal anomalies detection. dah: days after hatching. Different numbers (with the same prefix) refer to each replicate from the same enrichment product.

Dietary Replicates	EA-1	EA-2	EB-1	EB-2	EC-1	EC-2	ED-1	ED-2
Sampled individuals at 31 dah	26	25	25	25	25	20	35	24
Sampled individuals at 105 dah	26	24	26	25	26	25	25	25

Table S2. Typology of skeletal anomalies that were considered in this study for each anatomical region. The anomaly types were merged in anomaly categories according to the body region and the affected bony elements (code name is between parentheses). A: abdominal region; C: caudal region; CC: caudal complex region; VBA: vertebral body anomalies; VCD: vertebral column deviations.

Region	Skeletal Elements	Anomaly Typology	Anomaly Categories (Code)
Abdominal region	Parapophysis	Bifurcation<br>Number alteration<br>Insertion alteration<br>Fusion<br>Deformation	Parapophysis(PP)
	Neural arches and spines	Bifurcation<br>Number alteration<br>Insertion alteration<br>Fusion<br>Incomplete arch<br>Deformation	A Neural elements (A-N)
	VBA	Fusion	A Fusion (A-F)
		Deformation	A Deform (A-D)
	VCD	Kyphosis	A Kyphosis (A-K)
		Lordosis	A Lordosis (A-L)
		Scoliosis	A Scoliosis (A-S)
Caudal region	Neural arches and spines	Bifurcation<br>Number alteration<br>Insertion alteration<br>Fusion<br>Incomplete arch<br>Deformation	C Neural elements (C-N)
	Haemal arches and spines	Bifurcation<br>Number alteration<br>Insertion alteration<br>Fusion<br>Incomplete arch<br>Deformation	C Haemal elements (C-H)
	VBA	Fusion	C Fusion (C-F)
		Deformation	C Deform (C-D)
	VCD	Kyphosis	C Kyphosis (C-K)
		Lordosis	C Lordosis (C-L)
		Scoliosis	C Scoliosis (C-S)
Caudal complex	Neural arches and spines	Bifurcation<br>Number alteration<br>Insertion alteration<br>Fusion<br>Incomplete arch	CC Neural elements (CC-N)

Haemal arches and spines	Deformation<br>Bifurcation<br>Number alteration<br>Insertion alteration<br>Fusion<br>Incomplete arch	CC Haemal elements (CC-H)
Hypurals, epural, parhypural	Deformation<br>Bifurcation<br>Number alteration<br>Insertion alteration<br>Fusion<br>Deformation	Hypurals, epural and parhypural (HYE)
VBA	Fusion<br>Deformation	CC Fusion (CC-F)<br>CC Deform (CC-D)
VCD	Kyphosis<br>Lordosis<br>Scoliosis	CC Kyphosis (CC-K)<br>CC Lordosis (CC-L)<br>CC Scoliosis (CC-S)

Table S3. Fish density (number of fish/L) for each replicate tank (EA-1, EA-2, EB-1, EB-2, EC-1, EC-2, ED-1, ED-2) at different points of the experiment. dah: days after hatching. Different numbers (with the same prefix) refer to each replicate from the same enrichment product.

Age	EA-1	EA-2	EB-1	EB-2	EC-1	EC-2	ED-1	ED-2
1 dah (estimated)	20	20	20	20	20	20	20	20
15 dah	108	103	95	94	94	84	69	95
28 dah	20	19	18	18	18	16	13	18
105 dah	16	15	13	13	13	12	9	14

Table S4. Daily survival rate (%) from 15 to 105 days after hatching (dah) for each replicate tank (EA-1, EA-2, EB-1, EB-2, EC-1, EC-2, ED-1, ED-2): mean ± standard error of the mean, median, minimum and maximum. Different numbers (with the same prefix) refer to each replicate from the same enrichment product.

Survival rate		EA-1	EA-2	EB-1	EB-2	EC-1	EC-2	ED-1	ED-2
Survival rate (%)	Mean ± SEM	82.9 ±.0.8	83.0 ± 0.8	80.8 ± 0.9	80.2 ± 0.9	81.1 ± 0.8	79.4 ± 0.9	74.2 ± 1.1	81.1 ± 0.8
	Median	79.7	79.9	77.3	76.5	78.0	76.1	69.3	77.8
	Minimum	76.2	76.3	73.1	72.2	74.1	72.1	64.6	74.3
	Maximum	100.0	100.0	100.0	100.0	100.0	100.0	100.0	100.0
